# Non-alcoholic fatty liver disease: a narrative review of genetics

**DOI:** 10.7555/JBR.32.20180045

**Published:** 2018-08-06

**Authors:** Christopher J. Danford, Zemin Yao, Z. Gordon Jiang

**Affiliations:** 1 Division of Gastroenterology and Hepatology, Beth Israel Deaconess Medical Center, Boston, MA 02215, USA; 2 Department of Biochemistry, Microbiology and Immunology, Ottawa Institute of Systems Biology, University of Ottawa, Ottawa, Ontario K1H 8M5, Canada.

**Keywords:** NAFLD, NASH, genetics, *PNPLA3*, *TM6SF2*, *GCKR*, *MBOAT7*

## Abstract

Non-alcoholic fatty liver disease (NAFLD) is now the most common cause of chronic liver diseases worldwide. It encompasses a spectrum of disorders ranging from isolated hepatic steatosis to nonalcoholic steatohepatitis (NASH), fibrosis, cirrhosis, and hepatocellular carcinoma. One of the key challenges in NAFLD is identifying which patients will progress. Epidemiological and genetic studies indicate a strong pattern of heritability that may explain some of the variability in NAFLD phenotype and risk of progression. To date, at least three common genetic variants in the *PNPLA3*, *TM6SF2*, and *GCKR* genes have been robustly linked to NAFLD in the population. The function of these genes revealed novel pathways implicated in both the development and progression of NAFLD. In addition, candidate genes previously implicated in NAFLD pathogenesis have also been identified as determinants or modulators of NAFLD phenotype including genes involved in hepatocellular lipid handling, insulin resistance, inflammation, and fibrogenesis. This article will review the current understanding of the genetics underpinning the development of hepatic steatosis and the progression of NASH. These newly acquired insights may transform our strategy to risk-stratify patients with NAFLD and to identify new potential therapeutic targets.

## Background

Non-alcoholic fatty liver disease (NAFLD) is the most common cause of chronic liver disease worldwide affecting up to 25% of the global population and a third of the US population^[1^–^3]^. Together with this growing epidemic, morbidity from NAFLD is on the rise with a 170% increase in cirrhosis caused by non-alcoholic steatohepatitis (NASH) on the liver transplant waitlist between 2004 and 2013 in the US. NASH cirrhosis is now the second leading indication for liver transplantation in the US^[4^–^5]^.

The NAFLD epidemic has gone hand-in-hand with its major risk factor- insulin resistance- manifested by increasing rates of obesity and type 2 diabetes mellitus^[6]^. One might consider human fatty liver simply an acquired trait due to over-eating, similar to the process required to produce *foie gras* in ducks. However, NAFLD is a heterogeneous disease ranging from isolated hepatic steatosis to nonalcoholic steatohepatitis and cirrhosis^[7]^ and progression seems to occur only in a subset of patients^[8^–^9]^. Growing evidence in the past decade points to a strong genetic contribution to both the development of NAFLD and its progression. Genetics show great promise in risk stratification and may lead to future therapeutic interventions^[10]^. This article seeks to summarize the current literature regarding NAFLD genetics and their potential utility in the management of NAFLD patients.

## Heritability

A major observation indicating the heritability of NAFLD is the difference of its prevalence among ethnic groups. Two large multi-ethnic population studies in the US demonstrated that NAFLD was present at much higher rates in Hispanics compared to Caucasians, while African-Americans were relatively protected irrespective of insulin resistance and BMI^[2^,^11]^. Familial aggregation studies demonstrated that family members of overweight children with NAFLD were at higher risk of NAFLD compared to family members of overweight children without NAFLD^[12]^. Twin studies have also shown that up to 60% of the variability in serum ALT, a surrogate for liver fat content in the absence of alcohol or viral hepatitis, is genetically determined^[13]^. In another twin study using MRI proton-density fat fraction as a measurement of hepatic steatosis and MR elastography to determine hepatic fibrosis, both were highly correlated in monozygotic twins as compared to dizygotic twins^[4^,^11]^. After adjustment for age, ethnicity, and gender, the heritability of hepatic steatosis and hepatic fibrosis was 52% and 50%, respectively^[14]^.

Genome-wide association studies (GWAS) have been used in the past decade to define the specific genetic mediators of this heritability. An rs738409 C>G variant in the *patatin-like phospholipase domain-containing 3* (*PNPLA3*) gene, encoding an I148M mutation, was the first allele associated with intrahepatic fat content and appears to be a major genetic determinant of hepatic steatosis and the progression of fatty liver disease^[15^–^16]^. An rs58542926 C>T variant in the *transmembrane 6 superfamily member 2* (*TM6SF2*) gene, encoding an E167K mutation, and an rs780094 C>T variant in the *glucokinase regulator* (*GCKR*) gene were later shown to be associated with both hepatic steatosis and the risk of progression to fibrosis^[17^–^20]^. More recently, the rs641738 C>T variant of the *membrane bound O-acyltransferase domain-containing 7* (*MBOAT7*) gene was identified in alcoholic-related cirrhosis and subsequently confirmed to increase risk of hepatic steatosis and progressive liver disease in NAFLD^[21^–^22]^. Numerous other genetic variants involved in the pathogenesis of NAFLD, ranging from inflammatory response to insulin resistance and fibrogenesis, have also been implicated in NAFLD progression^[10]^ (***Table 1***).

**Tab.1:** Summary of genetic variants associated with NAFLD development and progression

Gene	Variant	Function	Variant effect	MAF	Hepatic phenotype	Extrahepatic phenotype
*PNPLA3*	rs738409 C>G	Lipid droplet remodeling	Impaired mobilization of FAs from lipid droplets through inhibition of other lipases, hepatic TG accumulation	0.267	↑NAFLD ↑NASH ↑fibrosis ↑HCC	↓CV risk No effect on IR
	rs2294918 G>A		Decreased PNPLA3 production, attenuation of effect of I148M variant	0.390	↓NAFLD ↓NASH	
*TM6SF2*	rs58542926 C>T	VLDL secretion	Decreased VLDL secretion, hepatic TG accumulation	0.067	↑NAFLD ↑NASH ↑fibrosis	↓CV risk No effect on IR
*GCKR*	rs780094 A>G rs1260326 C>T	Regulation of glucose influx to hepatocytes, *de novo* lipogenesis	Inability to regulate glucose influx into hepatocytes, increased *de novo* lipogenesis	0.302 0.293	↑NAFLD ↑NASH ↑fibrosis	No effect on CV risk ↓IR ↑CKD
*MBOAT7*	rs641738 C>T	Catalyzes acyl chain remodeling of phosphatidyl- inositols, reduces free arachidonic acid levels	Increased arachidonic acid levels, increased hepatic inflammation	0.440	↑NAFLD ↑NASH ↑fibrosis ↑HCC	No effect on CV or IR risk
*HSD17B13*	rs72613567:TA	Unknown. Localizes to hepatocyte lipid droplets.	Decreased HSD17B13 and PNPLA3 production	0.260	↓NASH ↓fibrosis	
*APOB*	multiple	VLDL secretion	Abetalipoproteinemia	0.01	↑NAFLD ↑NASH ↑fibrosis ↑HCC	↓CV risk
*MTTP*	multiple	VLDL secretion	Hypobetalipoproteinemia	0.01	↑NAFLD ↑NASH ↑fibrosis ↑HCC	↓CV risk
*LIPA*	multiple	Hydrolysis of cholesteryl esters and LDL particles	Lysosomal acid lipase deficiency	0.01	↑NAFLD ↑NASH ↑fibrosis	↑CV risk
*LPIN1*	rs13412852 C>T	Regulation of lipid metabolism	Reduced lipolysis, decreased flux of FAs to the liver	0.205	↓NASH ↓fibrosis	
*FATP5*	rs56225452 G>A	Hepatocyte FA uptake	Increased hepatocyte FA uptake	0.180	↑NASH	↑IR
*SOD2*	rs4880 C>T	Mitochondrial antioxidant	Increased oxidative stress	0.411	↑fibrosis	
*UCP2*	rs695366 G>A	Mitochondrial lipid metabolism	Increased UCP2 production, decreased oxidative stress	0.264	↓NASH	
*ENPP1*	rs1044498 A>C	Insulin signaling inhibitor	Increased inhibition of insulin signaling	0.342	↑fibrosis	↑IR
*IRS1*	rs1801279 A>C	Insulin signaling	Decreased insulin signaling	0.053	↑fibrosis	↑IR
*TRIB1*	rs2954021 G>A	Hepatic *de novo* lipogenesis	Increased hepatic TG		↑NAFLD	
*IL28B*	rs12979860 C>T	Innate immunity	Decreased IFN production	0.356	↓NASH ↓fibrosis	
*MERTK*	rs4374383 G>A	Innate immunity	Decreased hepatic stellate cell activation	0.360	↓fibrosis	↑IR
*KLF6*	rs3750861 G>A	Activation of HSCs	Decreased HSC activation	0.068	↓fibrosis	
*TERT*	multiple	Telomere maintenance	Accelerated hepatocyte senescence	0.01	↑fibrosis	

MAF: minor allele frequency; IR: insulin resistance; CV: cardiovascular; CKD: chronic kidney disease; HSC: hepatic stellate cell.

### PNPLA3

The *PNPLA3* gene encodes a 481-amino acid membrane protein located in the endoplasmic reticulum and lipid droplets in hepatocytes and hepatic stellate cells (HSCs)^[23]^. Transcription of *PNPLA3* is controlled by SREBP-1c and ChREBP, and is upregulated after feeding^[23^–^24]^. The PNPLA3 protein is post-transcriptionally modified by the presence of fatty acids to inhibit its degradation^[23^–^24]^. The rs738409 C>G polymorphism (I148M) in the *PNPLA3* gene, first identified by GWAS in 2008, encodes a missense mutation that by far is the most important genetic determinant for hepatic fat content^[15]^. The carrier frequency of *PNPLA3* I148M is as high as 49% in Hispanics, 23% in Caucasians, and 17% in African Americans in the Dallas Heart Study^[15]^. This genetic variant alone accounts for the ethnic difference in NAFLD prevalence.

*In vitro*, PNPLA3 acts as an acyltransferase that catalyzes the conversion of lysophosphatidic acid (LPA) to phosphatidic acid, while the I148M mutation renders a loss of enzymatic function^[25^–^26]^. However, the physiological function of PNPLA3 remains elusive. It is not clear whether the change in the enzyme activity contributes to the development of hepatic steatosis because *PNPLA3* knock-out mice do no develop hepatic steatosis^[26^–^28]^. Recent studies suggested that PNPLA3 may be involved in lipid droplet remodeling in hepatocytes, where the I148M variant protein accumulates on the surface of lipid droplets by evading ubiquitination and impairs hydrolysis of triglyceride by lipases^[26^,^29^–^31]^. This hypothesis is also supported by the finding that *PNPLA3* E434K, a variant that decreases *PNPLA3* expression, can attenuate the effect of I148M on hepatic steatosis and steatohepatitis^[32]^. Interestingly, the *PNPLA3* I148M variant is also present in HSCs, where the mutant allele is shown to activate HSCs independent of its role in hepatocytes^[33^–^34]^.

Clinically, aside from causing intrahepatic triglyceride accumulation, the *PNPLA3* I148M variant has also been shown to increase the risk of progressive liver disease. In the initial GWAS study by Romeo and coworkers, the I148M variant was associated with higher levels of alanine aminotransferase (ALT) indicating increased hepatic inflammation^[15]^. The *PNPLA3* I148M variant was subsequently found to be associated with NASH, hepatic fibrosis, and hepatocellular carcinoma (HCC)^[16^,^35^–^36]^. The *PNPLA3* I148M variant is also associated with increased risk of fibrosis progression and HCC in cirrhosis from hepatitis C and alcoholic liver disease independent of steatosis^[35^–^37]^, suggesting a potential direct contribution of the variant to fibrogenesis and carcinogenesis that are unrelated to intrahepatic triglyceride accumulation^[38]^.

### TM6SF2

The *TM6SF2* gene encodes a 351-amino acid protein with seven transmembrane domains expressed in the liver and intestine in humans^[39]^. The rs58542926 C>T (E167K) polymorphism in *TM6SF2* was first found to be associated with NAFLD by GWAS in 2014 and encodes a missense protein resulting in loss-of-function^[19]^. The *TM6SF2* E167K variant is present in 7.2% Europeans, 3.4% African Americans and 4.7% Hispanics. Although the function of TM6SF2 was not known at the time of this discovery, it was discovered shortly afterwards that the E167K variant impairs the lipidation and maturation of very low density lipoprotein (VLDL) in hepatocytes and chylomicrons in enterocytes, resulting in increased cellular triglyceride accumulation and decreased circulating triglyceride-rich lipoproteins^[19^,^39^–^40]^. Similar to the *PNPLA3* I148M variant, the *TM6SF2* E1267K variant not only increases the risk of steatosis, but has also been associated with increased risk of progressive liver disease and fibrosis^[20]^. This finding has also been observed in chronic hepatitis C patients with the *TM6SF2* E1267K allele^[41]^.

### GCKR

GCKR plays an important role in hepatic glucose uptake through regulating the partitioning of GCK (glucokinase) between the cytosol and nucleus^[42]^. The P446L (rs1260326 C>T) variant of *GCKR,* first identified by GWAS in 2011, encodes a loss-of-function protein unable to inhibit glucokinase in response to fructose-6-phosphate^[17^,^43]^. Thus, the P446L variant of *GCKR* is associated with increased hepatic glucose uptake, which in turn may contribute to increased *de novo* lipogenesis and concomitantly decreased serum glucose and insulin levels^[43]^. The combination of *PNPLA3* and *GCKR* minor alleles (referring to the less common variants, I148M and P446L, respectively) was shown to explain up to 30% of the liver fat content in obese children^[44]^. The *GCKR* P446L variant is also associated with an increased risk of fibrosis in NAFLD patients as well as elevated serum triglyceride levels^[18]^.

### MBOAT7

MBOAT7 (also known as lysophopshoplipid acyltransferase) catalyzes acyl chain remodeling of phosphatidylinositols, part of the Lands cycle, attaching arachidonic acid to lysophosphatidylinositol and reducing free arachidonic acid levels^[45]^. Arachidonic acid induces hepatocyte apoptosis, triggering hepatic inflammation and fibrosis^[46^–^47]^. Probably for this reason, the rs641738 C>T variant of *MBOAT7*, which results in decreased hepatic MBOAT7 expression, has not been observed to increase the risk of steatosis to the degree of *PNPLA3* or *TM6SF2*. Rather, the rs641738 C>T variant of *MBOAT7* is associated with an increase in hepatic inflammation and fibrosis in NAFLD^[21^,^48^–^49]^. The rs641738 C>T variant was first identified by GWAS in alcoholic liver disease in which it increases the risk of cirrhosis^[22]^, and has been implicated in hepatitis B^[50]^ and C^[51]^. Additionally, the rs641738 C>T variant has been tied to an increase in HCC risk in non-cirrhotics with NAFLD, as well as non-cirrhotic chronic hepatitis C and alcoholic liver disease^[52]^.

### HSD17B13

Most recently, exome-wide sequencing has identified the rs72613567:TA variant within *hydroxysteroid 17-beta dehydrogenase 13* (*HSD17B13*), which was associated with a decreased risk of alcoholic liver disease, NASH, alcoholic cirrhosis, and NASH cirrhosis^[53]^. The *HSD17B13* gene encodes a previously uncharacterized member of the hydroxysteroid 17-beta dehydrogenase family, and is primarily expressed in hepatocytes where the protein product is localizes to lipid droplets^[54]^. The precise function of HSD17B13 is currently unknown, but the splice variant results in *in vitro* loss of enzymatic function towards estradiol^[53]^. In a recent study of Abul-Husn *et al.*, the rs72613567:TA variant was associated with a 30% decreased risk of NASH and a 49% decreased risk of NASH cirrhosis without an association with hepatic steatosis itself, suggesting a role in mitigating liver injury but not a role in intrahepatic triglyceride accumulation. Further study is required to confirm this association and the role of HSD17B13 in NAFLD pathogenesis.

## Hepatic lipid metabolism

While *PNPLA3* and *TM6SF2* appear to be the most prominent population-wide determinants of hepatic steatosis, other relatively rare or less prominent genetic defects in intrahepatic lipid metabolism have been shown to cause fatty liver. Mutations in the genes governing hepatic processing and secretion of VLDL have been implicated in familial causes of NAFLD^[55]^. For instance, mutations within the *apolipoprotein B* (*APOB*) or *proprotein convertase subtilisin kexin 9* (*PCSK9*) gene result in familial hypobetalipoproteinemia, characterized by low or absent plasma levels of apoB and LDL-C^[56]^. The apoB protein is responsible for the assembly and secretion of hepatic VLDL and intestinal chylomicrons. Loss-of-function or missense mutations within the *APOB* gene can result in a decrease in serum cholesterol and an increase in intrahepatic triglycerides that have been implicated in familial cases of steatohepatitis, cirrhosis, and hepatocellular carcinoma^[57]^.

PCKS9 is a serine protease that enhances the degradation of LDL-receptors. Loss-of-function mutations within *PCKS9* similarly result in decreased serum cholesterol, but do not seem to be associated with increased hepatic triglycerides^[58]^. Large phase Ⅲ clinical trials investigating monoclonal antibodies against PCSK9 to lower serum LDL similarly have not demonstrated any adverse hepatic side effects^[59^–^60]^, though *PCSK9* mutations may exacerbate liver disease in those with *PNPLA3* and *TM6SF2* mutations^[61]^.

The *microsomal triglyceride-transfer protein* (*MTTP*) gene encodes a lipid transfer protein responsible for the recruitment of triglycerides during VLDL particle formation within the endoplasmic reticulum and may also serve as a chaperone assisting the folding of apoB in hepatocytes^[62]^. Mutations within the *MTTP* gene are responsible for abetalipoproteinemia, characterized by nearly undetectable plasma levels of LDL and apoB, as well as accumulation of triglycerides in the liver with subsequent cirrhosis^[63^–^64]^. Lomitapide is an inhibitor of MTTP marketed for the treatment of familial hypertriglyceridemia and acute pancreatitis. Long-term use of lomitapide has been associated with NASH cirrhosis^[65]^.

Apolipoprotein C3 (apoC3) is a major constituent of VLDL particles and promotes triglyceride-rich VLDL assembly and secretion^[66]^. Several mutations within the *APOC3* gene promoter have been associated with hypertriglyceridemia^[67^–^68]^, and initial reports also indicated increased risk of fatty liver^[69]^. However, subsequent studies have failed to find an association between polymorphisms within the *APOC3* promoter and progressive NAFLD^[70^–^71]^. Several variants within the coding sequence of apoC3 have been associated with hypotriglyceridemia^[72^–^73]^ and hypertriglyceridemia^[74]^, respectively. The relationship between these apoC3 coding sequence mutations and NAFLD is unclear. However, a recent study showed expression of the Gln38Lys variant in mice resulted in hepatic steatosis^[75]^. Since inhibition of apoC3 expression is currently being considered as a treatment of hypertriglyceridemia, one must keep in mind the potential risk of NAFLD and NASH^[76]^.

Another rare familial cause of NASH cirrhosis is the deficiency of lysosomal acid lipase (LIPA), an autosomal recessive lysosomal storage disease caused by loss-of-function mutations within the *LIPA* gene. Lysosomal acid lipase is responsible for hydrolyzing cholesteryl esters, triglycerides, and LDL particles into free cholesterol and fatty acids. Its deficiency frequently results in death in infancy, though in adults it leads to hypercholesterolemia, cardiovascular disease, hepatic steatosis, and cirrhosis^[77]^.

Fatty acid transport proteins (FATP) mediate hepatocyte uptake of free fatty acids. FATP5 increases hepatic free fatty acid uptake and silencing of FATP5 reverses steatosis in diet-induced NAFLD mice^[78^–^79]^. A variant (rs56225452) in the *FATP5* promoter region regulating gene expression was associated with increased hepatic steatosis, higher ALT levels, and increased insulin resistance in the general population^[80]^.

*LPIN1* encodes a phosphatidate phosphatase expressed in adipose tissue and the liver where it acts as an inducible transcriptional coactivator to regulate fatty acid metabolism^[81]^. The rs12412852 polymorphism of *LPIN1* was associated with a lower prevalence of NASH and hepatic fibrosis in a cohort of Italian children with NAFLD^[82]^. The mutation is thought to result in reduced lipolysis creating decreased flux of free fatty acids to the liver^[82]^.

## Oxidative stress and inflammatory response

Reactive oxidative species (ROS) produced with fatty acid beta oxidation in the mitochondria has been proposed as a major driver for NASH^[83]^. Several variations in genes involved in regulating mitochondrial redox status have been implicated in NASH progression. Uncoupling protein 2 (UCP2) regulates mitochondrial redox status by uncoupling oxidative phosphorylation^[84]^. A variation in the *UCP2* promoter region (rs695366) increases UCP2 expression and is associated with decreased risk of NASH in patients with normal fasting glucose^[85]^. Similarly, another protein involved in mitochondrial protection from oxidative stress, the mitochondrial manganese-dependent superoxide dismutase (MnSOD), has been associated with protection from progressive NAFLD^[86]^. The rs4880 polymorphism in the *SOD2* gene results in an increase in enzyme activity and was associated with a lower risk of NASH and fibrosis^[86]^.

In addition to ROS activation, NASH is associated with activation of the innate immune response due to sterile inflammation and gut-derived bacterial products^[87]^. Interferon-λ3/λ4 (encoded by the *IL28B* gene) was first noted to be involved in hepatic innate immunity when the rs12979860 CC variant was associated with increased clearance of hepatitis C virus^[88]^. This variant leads to increased production of Interferon-λ3 and has been associated with increased steatohepatitis and fibrosis in NAFLD^[89^–^90]^.

Polymorphisms in the promoter region of the *TNFA* gene encoding tumor necrosis factor-α have also been associated with the progression of NAFLD^[91^–^92]^, though results have not been consistent^[93^–^94]^ and may be due to linkage disequilibrium with other genes in the major human histocompatibility complex region^[10]^.

*MERTK* encodes a tyrosine kinase that initiates the removal of dying cells by phagocytes and is involved in the activation of HSCs. The rs4374383 non-coding variant reduces MERTK expression and is associated with reduced fibrosis in both NAFLD and chronic hepatitis C infection^[95^–^97]^.

## Insulin resistance

Insulin resistance is closely associated with NAFLD pathophysiology and disease progression^[98]^. Outside of environmental factors that contribute to insulin resistance, genetic variations in the insulin signaling pathway have also been linked to NAFLD progression and fibrosis. In hepatocytes, insulin binds to the insulin receptor resulting in the activation of insulin receptor substrate-1 (IRS-1) and decreases glucose production. A loss-of-function mutation (rs1801278) in *IRS1* results in hyperglycemia and has been associated with increased hepatic fibrosis^[99]^. A gain-of-function mutation in *ectonucleotide pyrophosphatase/phosphodiesterase1* (*ENPP1*) inhibits insulin receptor activity, resulting in insulin resistance and accelerated liver fibrosis in a cohort of obese NAFLD patients^[99]^.

Tribbles homolog1 (TRIB1) is a protein kinase involved in hepatic lipogenesis and glycogenesis^[100]^. Induction of TRIB1 expression increases plasma glucose and hepatic triglyceride production in a mouse model^[101]^. In humans, the rs2954021 polymorphism within *TRIB1* has been associated with increased plasma triglycerides and the development of NAFLD^[102]^.

## Fibrogenesis

Several genetic polymorphisms associated with cell senescence have also been associated with fibrosis in NAFLD. For example, telomeres, which serve to prevent DNA damage and cell senescence with cell division, are maintained by the enzyme telomerase. Loss-of-function mutations in the telomerase reverse transcriptase (*TERT*) gene are associated with familial liver disease and accelerated development of cirrhosis and HCC in NAFLD and other etiologies of chronic liver disease^[103^–^104]^. In the same vein, p21 regulates the cell cycle by causing cell cycle arrest and cell senescence. The rs762623 SNP in the *CDKN1A* gene, which encodes p21, has been associated with progressive NAFLD and fibrosis in a cohort of NAFLD patients^[105]^.

Hepatocyte senescence leads to hepatic fibrosis through the activation and proliferation of HSC^[106]^. Krueppel-like factor 6 (KLF6) is expressed by activated HSCs after liver injury resulting in collagen α1 transcription^[107]^. The rs3750861 variant of *KLF6* results in alternative splicing and decreased activation of HSC after liver injury and reduced fibrosis^[108]^. In addition, the rs3750861 variant of *KLF6* has been associated with decreased hepatic insulin resistance^[109]^.

Hepatic iron deposition promotes fibrogenesis through multiple pathways including oxidative stress, subsequent mitochondrial dysfunction^[110]^, and direct stimulation of HSC^[111]^. Genetic variations in the *HFE*, *beta-globin*, and *TMPRSS6* genes predispose to hepatic iron accumulation and are associated with hepatic fibrosis in NAFLD patients^[112^–^114]^.

## Extrahepatic manifestations of NAFLD

In addition to modulating the hepatic phenotype of NAFLD, genetic variations play an important role in extrahepatic manifestations of the disease. NAFLD is an independent risk factor for the development of cardiovascular disease, the leading cause of death in NAFLD patients^[115]^. Given the intimate involvement of many NAFLD-related genetic variants in lipid and lipoprotein metabolism, it follows that these variations may also affect cardiovascular risk. Indeed, the *TM6SF2* minor allele (*ie* E167K) which results in decreased hepatic VLDL secretion is associated with a lower cardiovascular risk, while the major allele increases total cholesterol and higher risk of myocardial infarction^[116^–^117]^. The *PNPLA3* I148M variant, which has a somewhat lesser effect on plasma lipids, has also been associated with a degree of protection from cardiovascular disease^[117^–^118]^. The *MBOAT7* minor allele, in which no effect on plasma lipids has been observed^[21]^, also has a neutral effect on cardiovascular risk^[117]^. The *GCKR* rs1260326 variant has been shown to increase plasma triglycerides with no change in LDL or HDL levels and has no effect on cardiovascular risk^[119]^. Lysosomal acid lipase deficiency results in both increased serum cholesterol and hepatic triglycerides, greatly increasing cardiovascular risk^[120]^. While coronary artery disease is the leading cause of death for all-comer NAFLD patients, the genetics of NAFLD impact the cardiovascular risk in different ways, and a better understanding of an individual’s genetic underpinnings may also help to stratify their cardiovascular risk.

While acquired risk factors such as obesity lead to insulin resistance and both NAFLD and type 2 diabetes, any relationship beyond this is less clear. Similar to cardiovascular risk, when disentangling the link between NAFLD and type 2 diabetes, it is helpful to turn to genetics and the mechanism by which these genes exert their effects. For example, the minor alleles of both *PNPLA3* and *TM6SF2* result in hepatocyte lipid retention without any direct link to insulin signaling or glucose metabolism. Accordingly, neither the I148M nor E167K variant is associated with increased insulin resistance or incidence of type 2 diabetes^[15^,^19^,^40^,^121]^. Similarly, *MBOAT7* that regulates circulating phosphatidylinositol composition, has no impact on insulin resistance or type 2 diabetes prevalence^[21]^. The *GCKR* P446L variant, on the other hand, results in the deregulation of hepatic glucose uptake, increased *de novo* lipogenesis, decreased serum glucose, and reduced insulin resistance^[44^,^122]^. Polymorphisms in the *ENPP1* and *IRS1* genes involved in insulin signaling result in insulin resistance and are associated with fibrosis in NAFLD^[99]^ as well as risk of incident diabetes^[123^–^124]^. While NAFLD is often driven by insulin resistance, genetics predisposing to NAFLD largely do not impact insulin resistance directly.

NAFLD is also associated with chronic kidney disease^[125]^. The *GCKR* rs1260326 polymorphism has been associated with increased risk of chronic kidney disease in those with NAFLD, though the mechanism is not clear and results have not been replicated^[126]^.

Genetic variations have proven useful in understanding not only the phenotypic variability of liver disease in NAFLD, but also in the pathophysiology behind its comorbidities. Such an understanding may also prove useful in the risk stratification of NAFLD patients with shared extrahepatic comorbidities.

## Conclusions

**Fig.1:**
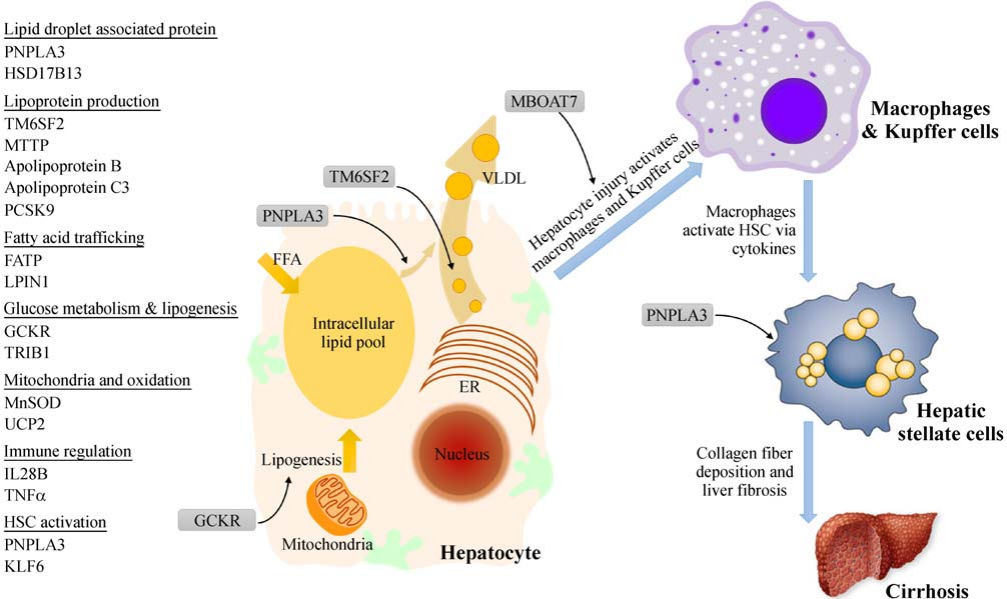
Genetic impact on hepatic steatosis, liver inflammation, and fibrosis in NAFLD.

Research in the past decade has shown that non-alcoholic fatty liver is a trait driven by both acquired and genetic causes, and has taught us important lessons. First of all, up to 50% of disease susceptibility and progression is heritable^[14]^. Major genetic determinants of hepatic steatosis in the population include *PNPLA3* I148M*, TM6SF2* E167K and *GCKR* P446L*.* These genes variances as well as less common genetic mutations linked to NAFLD highlights the importance of lipoprotein assembly, intrahepatic lipid handling, and glucose metabolism in the pathophysiology of NAFLD (***Fig. 1***). The genetics underlying the inflammation and fibrosis in NAFLD is less well-defined, mainly owing to a lack of large cohort studies with phenotyping and appropriate controls. Secondly, unfavorable genetics increases the disease susceptibility, but may not cause disease alone. This paradigm is most prominent in *PNPLA3*, where the I148M carriers are far more likely to develop NAFLD when they are obese, while lean carriers can still be disease-free. Last but not least, the presence of different genetic variances indicates that NAFLD is not one homogenous disease. Although they share the presence of fatty liver, their risk of progressive liver fibrosis and comorbidities may vary based on the genetic cause. This is exemplified by the impact on circulating lipoproteins by *TM6SF2* E167K and to a lesser extent *PNPLA3* I148M in NAFLD.

Understanding the genetic underpinnings of NAFLD provides an exciting opportunity to refine our management of NAFLD patients. Although the most recent AASLD guidelines do not recommend testing for genetic variants in routine clinical care^[127]^, the potential of such an approach has been demonstrated in several recent studies. Using genetics alone (*PNPLA3*, *SOD2*, *KLF6*, and *LPIN1*), one group was able to predict the presence of NASH in a cohort of children with NAFLD, though it was not useful in the prediction of fibrosis^[128]^. In one report, the minor allele of the *IL28B* gene along with clinical factors outperformed traditional non-invasive assessments in the prediction of fibrosis not just in NASH, but also in chronic viral hepatitis^[129]^. The combination of *PNPLA3* genotype, AST level, and fasting insulin level has been shown to be useful in predicting the histologic presence of NASH in a cohort of NAFLD patients^[130]^. Similarly, we have shown that a combination of *PNPLA3* and *TM6SF2* genotype along with a lipoprotein-derived assessment of insulin resistance was able to predict both NAFLD activity and fibrosis (unpublished data). As our understanding of the role of genetics in NAFLD pathophysiology improves, these models show great promise not only in the risk stratification of the hepatic consequences of NAFLD, but may also prove useful in disentangling the extrahepatic consequences as well.

Perhaps the most exciting aspect of NAFLD genetics is the potential therapeutic implications. Downregulation of *PNPLA3* I148M variant production by the E434K variant has been shown to attenuate the effect of the I148M variant on steatohepatitis^[32]^. Though largely in the theoretical stage of development, downregulation of the *PNPLA3* I148M variant shows promise as a potential point of therapeutic intervention.

Genetic research over the past decade has provided valuable insight into the pathogenesis of NAFLD. Further refinement over the coming decade has the potential to transform the way we care for NAFLD patients with a personalized approach to monitoring tailored to an individual’s risk of fibrosis progression and treatment targeting the underlying mechanism of disease.
